# Supplementary material for: Bacterial diversity and community in Qula from the Qinghai–Tibetan Plateau in China
Source: PeerJ. 2018 Dec 5;6:e6044. doi: 10.7717/peerj.6044 (PMC6286660; doi:10.7717/peerj.6044)
Supplement: Supplemental Information 1 — FTU: fraction of unexplained taxonomic units. Letters indicate Duncan’s pairwise differences among samples from different origins (P < 0.05). [file peerj-06-6044-s001.docx]

Supplementary Table 1: The fraction of unexplained taxonomic units per sample and averaged per location. FTU: fraction of unexplained taxonomic units. Letters indicate Duncan’s pairwise differences among samples from different origins (P < 0.05).

| Sample ID | FTU (%) | Average FTU per location (%) |
| --- | --- | --- |
| YN1 | 3.79 |  |
| YN2 | 3.35 | 3.46±0.29^e^ |
| YN3 | 3.24 |  |
| XZ1 | 9.31 |  |
| XZ2 | 9.37 | 9.21±0.22^b^ |
| XZ3 | 8.96 |  |
| QH1 | 9.61 |  |
| QH2 | 4.58 | 6.55±2.68^bc^ |
| QH3 | 5.47 |  |
| GN1 | 5.92 |  |
| GN2 | 7.60 | 6.40±1.04^cd^ |
| GN3 | 5.69 |  |
| SC1 | 16.05 |  |
| SC2 | 14.23 | 14.35±1.64^a^ |
| SC3 | 12.77 |  |
