# Supplementary figures and images for: Bacterial diversity and community in Qula from the Qinghai–Tibetan Plateau in China
Source: PeerJ. 2018 Dec 5;6:e6044. doi: 10.7717/peerj.6044 (PMC6286660; doi:10.7717/peerj.6044)

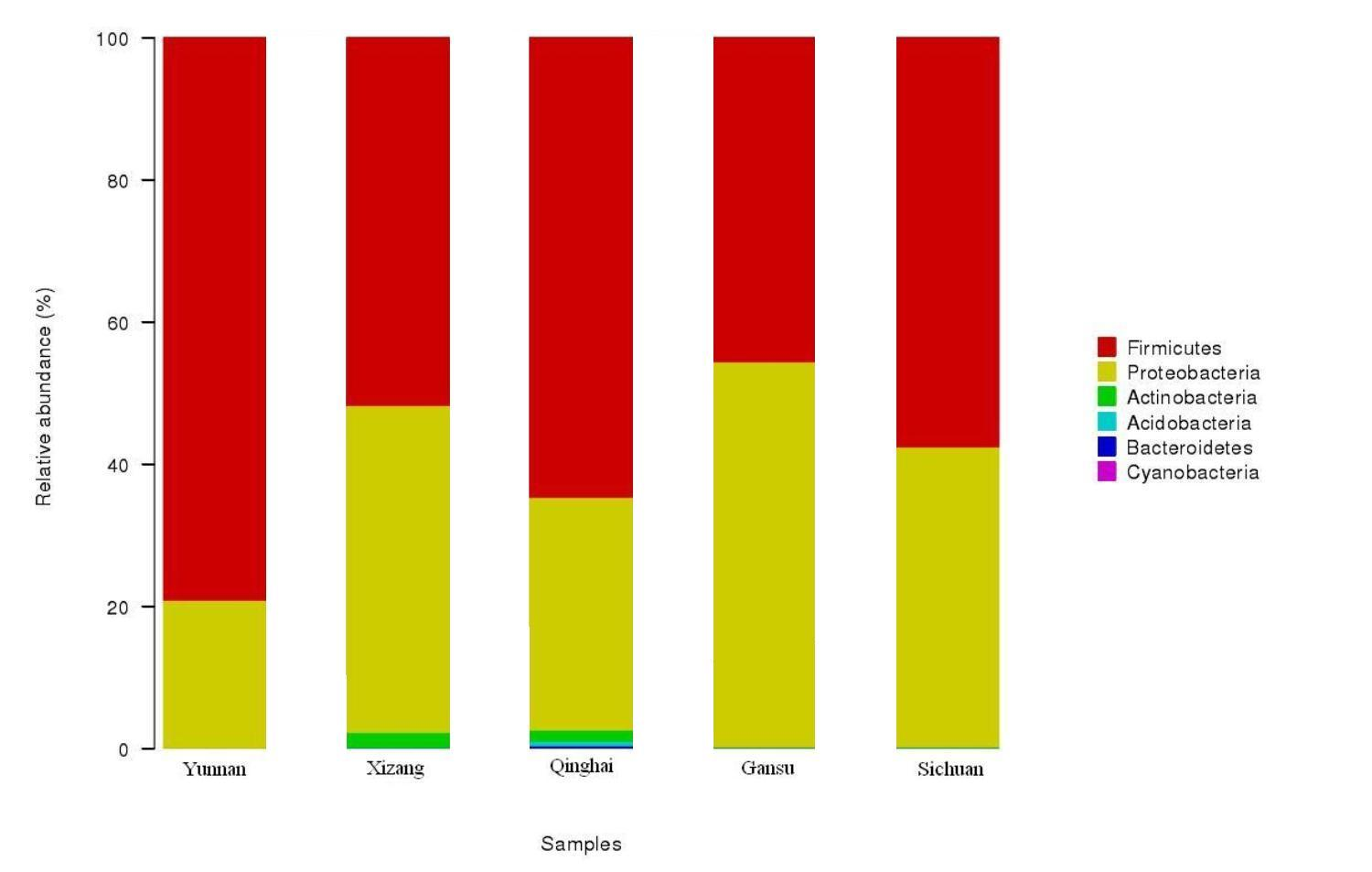

Supplement: Supplemental Information 2 [file peerj-06-6044-s002.png]

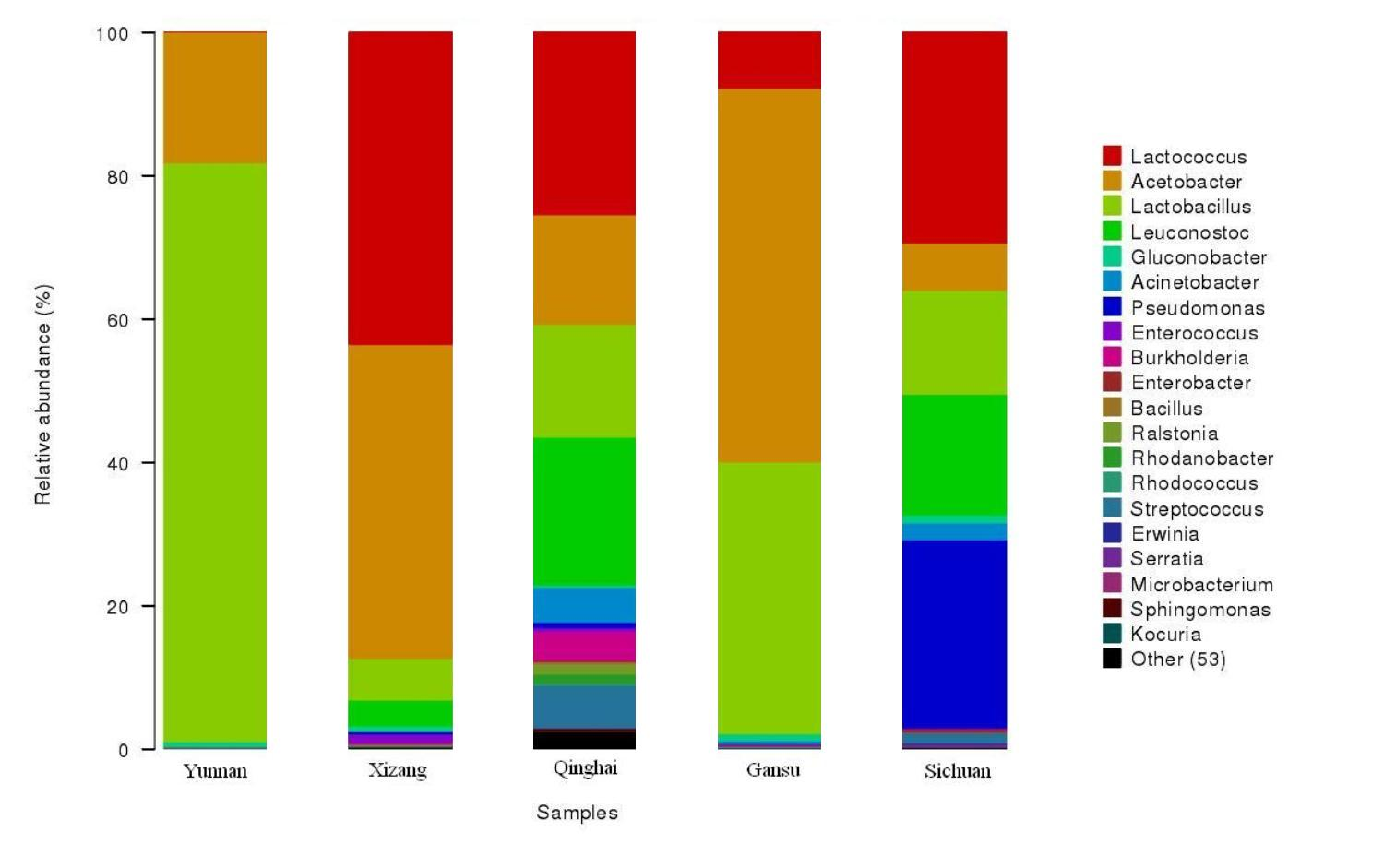

Supplement: Supplemental Information 3 [file peerj-06-6044-s003.png]
